# Hakuna mycotic aneurysm, *Streptococcus salivarius* does not always mean “no worries”

**DOI:** 10.1016/j.amsu.2021.102798

**Published:** 2021-09-04

**Authors:** Saad Ahmad, David Song, Jonathan Vincent M. Reyes, Adrian Whiting, Talal Almas, Joseph J. Lieber

**Affiliations:** a Department of Internal Medicine, Icahn School of Medicine at Mount Sinai Hospital (Elmhurst Hospital Center) NY, USA; b Department of Internal Medicine, NYU Langone Hospital - Long Island, Mineola, NY, USA; c Royal College of Surgeons in Ireland, Dublin, Ireland; d Department of Nephrology, Icahn School of Medicine at Mount Sinai Hospital (Elmhurst Hospital Center) NY, USA

**Keywords:** Mycotic aneurysm, Endocarditis, Viridans, Streptococcus, Duke criteria, Bacteremia

## Abstract

*Streptococcus salivarius*, an easily missed and commonly disregarded Viridians strep species, is usually written off as a culture contaminant, but has been implicated as a rare cause of bacterial endocarditis with serious complications. It is a normal commensal microorganism of the mouth and gut, *S. salivarius* is usually harmless and even demonstrates anti-inflammatory properties. However, the literature about the complications of a *S. salivarius* bacteremia remains unclear. This case highlights a patient with mycotic aneurysms due to infective endocarditis in the setting of *S. salivarius* bacteremia.

## Highlights

• *Streptococcus salivarius* is easily missed and usually a culture contaminant, but it is also a rare cause of bacterial endocarditis with serious complications.
• Infective endocarditis is diagnosed based on the modified Duke's criteria and commonly presents with a low-grade fever, a new onset heart murmur, petechiae, subungual hemorrhages, Janeway lesions, Osler nodes, or Roth spots.
• It is critical to recognize *S. salivarius*'s role in bacterial endocarditis and its serious complications; therefore, rapid identification and treatment with antibiotics are imperative to avoid complications.

## 1 Introduction

*Streptococcus salivarius*, a member of the Viridans streptococci family, is a commensal bacterium of the human oral mucosa and gut, and often considered a contaminant and an uncommon source of bacteremia. As one of the first colonizers of the oral cavity and gut after birth, *S. salivarius* plays a major role in immune system homeostasis and even contains anti-inflammatory properties [1,2]. While disseminated infection of *S. salivarius* is exceedingly rare, there have been many well-documented *S. salivarius* infections in the last decade demonstrating its capacity to affect the central nervous, cardiovascular, musculoskeletal, and gastrointestinal systems [3,4].

Viridans streptococci family accounts for roughly 40–60% of infective endocarditis cases [5]. However, *S. salivarius* as the causative agent comprises only a fraction of those cases. In one study, only 4 out of 183 cases of endocarditis (2%) were caused by *S. salivarius* [6]. The nidus for infective endocarditis is usually valvular heart disease such as mitral and aortic stenosis or regurgitation, but in the case of Viridans streptococci, it may also occur on normal heart valves. Viridans streptococci normally disseminate into the bloodstream and to the heart via dental lesions and form vegetations on the heart valves. Infective endocarditis is diagnosed based on the modified Duke's criteria **(**Table 1**)** and commonly presents with a low-grade fever (between 98.7 °F and 100.4 °F or 37.5 °C and 38.3 °C), a new onset heart murmur, and one or more of the following: petechiae, subungual hemorrhages, Janeway lesions, Osler nodes, or Roth spots.

**Table 1:** Modified Duke's infective endocarditis criteria [11,14].

Criteria	Definitive Infective Endocarditis	Possible Infective Endocarditis	Not Infective Endocarditis
Pathologic			
Histologic	Vegetation or intracardiac abscess present, confirmed by histology showing active endocarditis	Short of definite, but not rejected	No pathologic evidence of infective endocarditis with antibiotic therapy for 4 days or less
**OR**			
Bacteria	Demonstrated by culture or histology in a vegetation, or in a vegetation that has embolized, or in an intracardiac abscess	Short of definite, but not rejected	No pathologic evidence of infective endocarditis with antibiotic therapy for 4 days or less
**Clinical - Any one of the following**			
Major criteria	2	Does not apply	Resolution of manifestations of endocarditis, with antibiotic therapy for 4 days or less, or firm alternate diagnosis for manifestations of endocarditis. Does not meet criteria for possible infective endocarditis
Minor criteria	5	3	
Major and Minor criteria	1 major +3 minor	1 major and 1 minor	
Major criteria			
A. **Supportive laboratory evidence** Typical microorganism for infective endocarditis from two separate blood cultures: viridans streptococci, *Staphylococcus aureus*, Streptococcus bovis, HACEK group (Haemophilus spp. Actinobacillus actinomycetemcomitans, Cardiobacterium hominis, Eikenella spp., and Kingella kingae) or Community-acquired enterococci, in the absence of a primary focus			
Persistently positive blood culture, defined as recovery of a microorganism consistent with infective endocarditis from blood cultures drawn more than 12 hours apart or Persistently positive blood culture, defined as recovery of a microorganism consistent with infective endocarditis from all of three or a majority of four or more separate blood cultures, with first and last drawn at least 1 hour apart.			
Single positive blood culture for Coxiella burnetti or phase I antibody titer >1:800			
B. Evidence of endocardial involvement Echocardiogram supportive of infective endocarditis. 1. Type of study **TEE recommended as first test in the following patients**: a) prosthetic valve endocarditis; or b) those with at least “possible” endocarditis by clinical criteria; or c) those with suspected complicated endocarditis, such as paravalvular abscess. TTE recommended as first test in all other patients 2. **Definition of positive findings**: oscillating intracardiac mass, on valve or supporting structures, or in the path of regurgitant jets, or on implanted material, in the absence of an alternative anatomic explanation or myocardial abscess or new partial dehiscence of prosthetic valve			
C. New valvular regurgitation (increase or change in pre-existing murmur not sufficient).			
Minor Criteria			
Predisposing heart condition or Intravenous drug use			
Fever≥38C (100.4 F)			
Vascular phenomena: major arterial emboli, septic pulmonary infarcts, mycotic aneurysm, intracranial hemorrhage, conjunctival hemorrhage, Janeway lesions			
Immunologic phenomena: glomerulonephritis, Osler's nodes, Roth spots, rheumatoid factor			
Positive blood culture not meeting major criterion as noted previously (Excluding single positive cultures for coagulase-negative staphylococci and organisms that do not cause endocarditis) or serologic evidence of active infection with organism consistent with infective endocarditis			

*S. salivarius* is also known to cause the formation of mycotic aneurysms. A mycotic aneurysm is an aneurysm due to a disseminated infection that seeds the arterial intima and is associated with atherosclerotic arterial injury. Pro-inflammatory cytokines released due to the infection attract neutrophils which activate matrix metalloproteinases and degrade the vessel wall [7,8]. Presentation of mycotic aneurysms depends on the location of the aneurysm. In the case of cerebral mycotic aneurysms, critical presentations such as stroke and subarachnoid hemorrhage are not uncommon [[7], [8], [9]]. In addition, this work has been reported in accordance with SCARE [10].

## 2 Case presentation

A 73-year-old male with no significant past medical history presented with ten days of worsening bilateral hip, neck, and back pain that significantly limited his mobility. On arrival, the patient demonstrated stable vital signs and labs were notable for an elevated white blood cell count of 22,000 (4500 to 11,000 WBCs per microliter) with 93% neutrophils and an elevated troponin level to 1.369 ng/mL (0 and 0.4 ng/mL). Electrocardiogram (ECG) showed normal sinus rhythm with low voltage without evidence of T-wave inversion, ST segment elevation or depression, and the presence of Q-waves. Patient without any chest pain, palpitation, or shortness of breath.

Physical exam demonstrated normal dentition, high pitched, “blowing” holo-systolic murmur loudest at the apex, and tenderness upon the palpation of the paraspinal lumbar region. All other physical exam findings were otherwise unremarkable. The blood cultures identified *S. salivarius*, which was later confirmed with repeat culture. Given the sensitivities, the patient was started on a 6-week course of ceftriaxone and 2-week course of gentamicin. Magnetic resonance imaging (MRI) of the Lumbar spine demonstrated a likely L4-L5 osteomyelitis-discitis. Transthoracic echocardiogram (TTE) showed multiple enhanced lesions noted on the mitral and aortic valve concerning for vegetation and endocarditis. Transesophageal echocardiogram (TEE) was subsequently performed which demonstrated multiple vegetations on the mitral valve with severe mitral regurgitation and multiple vegetations on the aortic valve with severe aortic regurgitation. Cardiovascular surgery evaluated the patient for valve replacement, however deferred due to no signs and symptoms of heart failure.

**Fig. 1:**
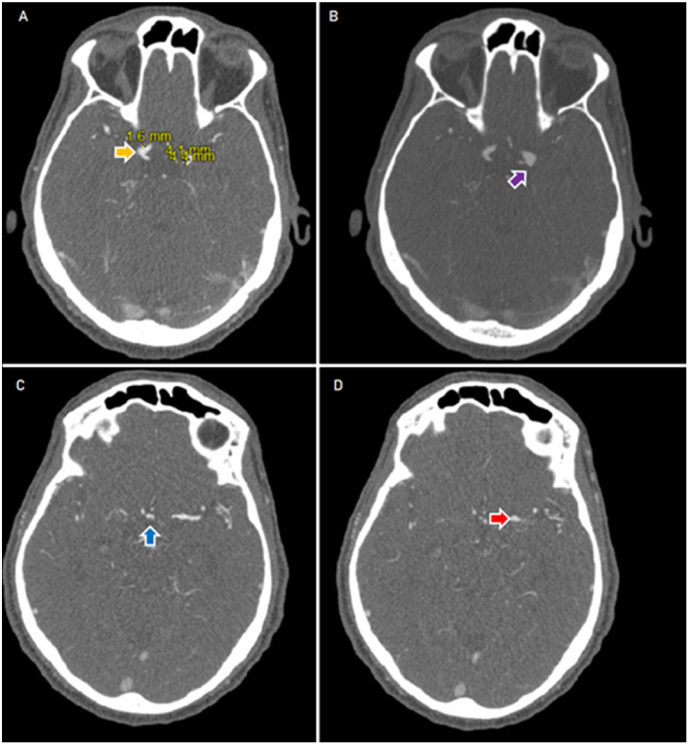
Computed Tomography angiogram demonstrating intracranial aneurysms in various anatomical locations. (A) Right terminal internal carotid artery (yellow), broad-based, measuring 1–2 mm projecting posteriorly (B) 4 × 4 mm broad-based left posterior communicating artery aneurysm (purple) projecting posteriorly (C) A 3 mm broad-based right anterior communicating artery aneurysm (blue) (D) A 3 mm broad-based aneurysm projecting superiorly from the left carotid bifurcations (red). (For interpretation of the references to colour in this figure legend, the reader is referred to the Web version of this article.)

The patient later was found to have a left lower extremity paralysis. Computed tomography (CT) scans of the brain without contrast, thoracic spine without contrast and lumbar spine without contrast did not demonstrate a clear cause of the acute weakness as the exams were unremarkable for acute pathology. Subsequent Brain MRI demonstrated an acute infarct of the left pons as well as multifocal moderate to severe intra and extracranial stenosis (bilateral vertebral, proximal basilar and left M1), and a 2mm anterior communicating artery aneurysm (ACOM). Follow-up computed tomography angiography (CTA) demonstrated four intracranial aneurysms (ACOM, right terminal carotid, left posterior communicating artery, and left carotid bifurcation) **(**Fig. 1**)**. The neurologists suspected subacute infarcts and the demonstration of mycotic aneurysms in the setting of infective endocarditis. Left lower extremity weakness was ultimately attributed to degenerative changes and inflammation causing nerve compression from osteomyelitis-discitis as noted on the MRI lumbar spine; imaging demonstrated grade 1 anterolisthesis of L4 over L5 with moderate thecal sac compression and bilateral neural foraminal narrowing at levels L4-L5 and L5-S1.

The patient was eventually discharged to rehab after completing a 14 day course of gentamicin and was to continue ceftriaxone for at least 6 weeks for infective endocarditis and L4-L5 osteomyelitis-discitis. During the rehabilitation course, the patient was noted to have a worsening acute kidney injury, likely due to gentamicin, and altered mental status. The patient was eventually sent back to the medicine service where they went into septic shock. Further workup up revealed multifocal pneumonia, shock liver, coagulopathy without evidence of disseminated intravascular coagulation and worsening congestive heart failure with persistent mitral valve and aortic valve vegetations. We believe the multiorgan failure was a result of late antibiotic intervention and resistance since the vegetations seemed to have grown since discharge to rehab. Unfortunately the patient expired as a result of his multiorgan failure.

## 3 Discussion

Only 5–15% of blood cultures that grow Viridans streptococci are *S. salivarius*, thus it is often regarded as a contaminant organism, especially since most cases have an unclear etiology [3]. There is no gold standard to differentiate between potential contamination and significant bacteremia in cases of blood culture isolation. It is important to recognize that our patient demonstrated two positive blood cultures of a Viridans group streptococci positive for endocarditis with evidence of endocardial involvement in both TTE and TEE. Utilizing the Duke criteria [11] as a diagnostic tool **(**Table 1**)**, our patient met both major criteria (two positive blood cultures for known endocarditis-causing bacteria and positive endocarditis on TEE) and at least one minor criterion (presence of mycotic aneurysms).

The presence of multiple intracranial aneurysms in the setting of a left-sided infective endocarditis points to the vascular phenomenon of intracranial mycotic aneurysms. Although mycotic aneurysms have a predilection for more peripheral branch points, approximately one-third of all bacterial intracerebral mycotic aneurysms can be proximally located, as demonstrated by our case [12]. Literature supporting the presence of mycotic aneurysms secondary to *S. salivarius* endocarditis in our patient states that the likelihood of endocarditis-associated mycotic aneurysms increases with age and in patients with underlying atherosclerotic disease [7]. In addition to these risk factors, the presence of an osteomyelitis-discitis allows the bacteria to navigate causing local and hematologic extension with involvement of the brain [7,13]. Finally, the temporal relationship between our patients's Duke criteria-supported diagnosis of bacterial endocarditis and the presence of mycotic aneurysms cannot simply be a coincidence. It is more plausible that our patient had chronic *S. salivarius* osteomyelitis that seeded to the bloodstream, formed vegetations in the heart, and migrated to his brain where it formed mycotic aneurysms.

## 4 Conclusion

*S. salivarius* is commonly considered a contaminant and is often disregarded. Our case highlights the real and potentially fatal complications of *S. salivarius*. It is our hope that clinicians will be informed by this case to recognize *S. salivarius*'s role in bacterial endocarditis and potential clinical sequelae if not properly treated; therefore, rapid identification and treatment with antibiotics are imperative to avoid complications secondary to bacteremia.

## Ethical approval

Research studies involving patients require ethical approval. Please state whether approval has been given, name the relevant ethics committee and the state the reference number for their judgement.

Obtained.

## Please state any sources of funding for your research

All sources of funding should be declared as an acknowledgement at the end of the text. Authors should declare the role of study sponsors, if any, in the collection, analysis and interpretation of data; in the writing of the manuscript; and in the decision to submit the manuscript for publication. If the study sponsors had no such involvement, the authors should so state.

None.

## Author contribution

Please specify the contribution of each author to the paper, e.g. study concept or design, data collection, data analysis or interpretation, writing the paper, others, who have contributed in other ways should be listed as contributors.SA, DS wrote the abstract, case presentation, study concept, images, conclusion.DS, JVR, AW reviewed paper, wrote discussion.DS, TA, JL performed final edits.

## Please state any conflicts of interest

All authors must disclose any financial and personal relationships with other people or organisations that could inappropriately influence (bias) their work. Examples of potential conflicts of interest include employment, consultancies, stock ownership, honoraria, paid expert testimony, patent applications/registrations, and grants or other funding.

None.

## Registration of research studies

In accordance with the Declaration of Helsinki 2013, all research involving human participants has to be registered in a publicly accessible database. Please enter the name of the registry and the unique identifying number (UIN) of your study.

You can register any type of research at http://www.researchregistry.com to obtain your UIN if you have not already registered. This is mandatory for human studies only. Trials and certain observational research can also be registered elsewhere such as: ClinicalTrials.gov or ISRCTN or numerous other registries.

1. Name of the registry: NA
2. Unique Identifying number or registration ID: NA
3. Hyperlink to your specific registration (must be publicly accessible and will be checked): NA

## Provenance and peer review

Not commissioned, externally peer reviewed.

## Acknowledgement

None.

## Consent

Studies on patients or volunteers require ethics committee approval and fully informed written consent which should be documented in the paper.

Authors must obtain written and signed consent to publish a case report from the patient (or, where applicable, the patient's guardian or next of kin) prior to submission. We ask Authors to confirm as part of the submission process that such consent has been obtained, and the manuscript must include a statement to this effect in a consent section at the end of the manuscript, as follows: “Written informed consent was obtained from the patient for publication of this case report and accompanying images. A copy of the written consent is available for review by the Editor-in-Chief of this journal on request”.

Patients have a right to privacy. Patients’ and volunteers' names, initials, or hospital numbers should not be used. Images of patients or volunteers should not be used unless the information is essential for scientific purposes and explicit permission has been given as part of the consent. If such consent is made subject to any conditions, the Editor in Chief must be made aware of all such conditions.

Even where consent has been given, identifying details should be omitted if they are not essential. If identifying characteristics are altered to protect anonymity, such as in genetic pedigrees, authors should provide assurance that alterations do not distort scientific meaning and editors should so note.

Obtained.

## Guarantor

The Guarantor is the one or more people who accept full responsibility for the work and/or the conduct of the study, had access to the data, and controlled the decision to publish.

## Declaration of competing interest

None.

The following information is required for submission. Please note that failure to respond to these questions/statements will mean your submission will be returned. If you have nothing to declare in any of these categories then this should be stated.
